# Bacterial c-di-GMP triggers metamorphosis of mussel larvae through a STING receptor

**DOI:** 10.1038/s41522-024-00523-7

**Published:** 2024-06-20

**Authors:** Xiao-Meng Hu, Lihua Peng, Yuyi Wang, Fan Ma, Yu Tao, Xiao Liang, Jin-Long Yang

**Affiliations:** 1 International Research Center for Marine Biosciences, Ministry of Science and Technology, Shanghai Ocean University, Shanghai, China; 2 Shanghai Collaborative Innovation Center for Cultivating Elite Breeds and Green-Culture of Aquaculture Animals, Shanghai, 201306 China

**Keywords:** Symbiosis, Biofilms

## Abstract

Bacteria induced metamorphosis observed in nearly all marine invertebrates. However, the mechanism of bacteria regulating the larvae-juvenile metamorphosis remains unknown. Here, we test the hypothesis that c-di-GMP, a ubiquitous bacterial second-messenger molecule, directly triggers the mollusc *Mytilus coruscus* larval metamorphosis via the stimulator of interferon genes (STING) receptor. We determined that the deletion of c-di-GMP synthesis genes resulted in reduced c-di-GMP levels and biofilm-inducing activity on larval metamorphosis, accompanied by alterations in extracellular polymeric substances. Additionally, c-di-GMP extracted from tested varying marine bacteria all exhibited inducing activity on larval metamorphosis. Simultaneously, through pharmacological and molecular experiments, we demonstrated that *M. coruscus* STING (*Mc*STING) participates in larval metamorphosis by binding with c-di-GMP. Our findings reveal that new role of bacterial c-di-GMP that triggers mussel larval metamorphosis transition, and extend knowledge in the interaction of bacteria and host development in marine ecosystems.

## Introduction

Biofilms are one of the oldest and most successful forms of life on Earth^1^, widely distributed across the globe. They can be regarded as biogenic habitat formers^2^, where bacteria gather to secrete extracellular polymeric substances (EPS), creating a relatively stable community structure^3^. In the oceans, this structure forms a habitat for other bacteria, algae, and marine invertebrates, providing essential survival information for other organisms^4^.

Invertebrates larvae need undergo a transformation from planktonic larva to benthic adult, a process referred to as settlement and metamorphosis^5^. Bacteria are known to stimulate metamorphosis in various animal groups, including mussel *Mytilus coruscus*^6–9^. However, not all bacteria can trigger this transformation. Huang et al. discovered that subtle variations among strains of the same bacterium played a key role in their ability to induce metamorphosis^10^. Therefore, in the last decade, researchers initiated studies to identify the gene(s) responsible for inducing settlement in these bacterial strains^6^. Previous studies in our laboratory have revealed that deletion of polysaccharide synthesis genes results in reduced biofilm attractiveness to larvae^11,12^. Remarkably, during this process, bacterial c-di-GMP regulated the secretion of these EPS and finally impacted the larval metamorphosis rate^11–13^. However, the efficiency and mechanism of c-di-GMP in controlling larval recruitment is unknown.

C-di-GMP, a ubiquitous second messenger in bacteria^14^, plays a crucial role in facilitating the transition of bacteria from a planktonic state to adopting a biofilm lifestyle^15^. It is associated with many physiological processes in bacteria, including sensing environmental cues, regulating polysaccharide biosynthesis, modulating bacterial motility, and playing a central role in biofilm dispersal^16^. However, there is still limited knowledge about the how bacterial c-di-GMP pathways impact bacterial physiological traits, especially larval recruitment.

In this study, we selected a strain of *Pseudoalteromonas marina* with a stable moderate inducibility for larval metamorphosis to investigate the effects of c-di-GMP synthesis gene on biofilm properties and larval recruitment capacity^17^. The larval recruitment of c-di-GMP was further confirmed by pharmacological experiments. Additionally, the stimulator of interferon genes (STING) was validated as a receptor combing to c-di-GMP through molecular interaction experiments. We used RNA interference technique to silence the *M. coruscus STING* (*McSTING*) gene and identified the involvement of *McSTING* in larval metamorphosis. Given the ubiquity of c-di-GMP in bacteria, this study underscores the potential of c-di-GMP as a universal inducer of metamorphosis in marine invertebrates. This study has established a signaling pathway that bridges bacteria and larval metamorphosis and offers invaluable insights in both invertebrate development and the interactions between microorganisms and their hosts.

## Results

### Reduction of larval metamorphosis by Δ*cdg* biofilm with lower c-di-GMP contents

**Fig. 1:**
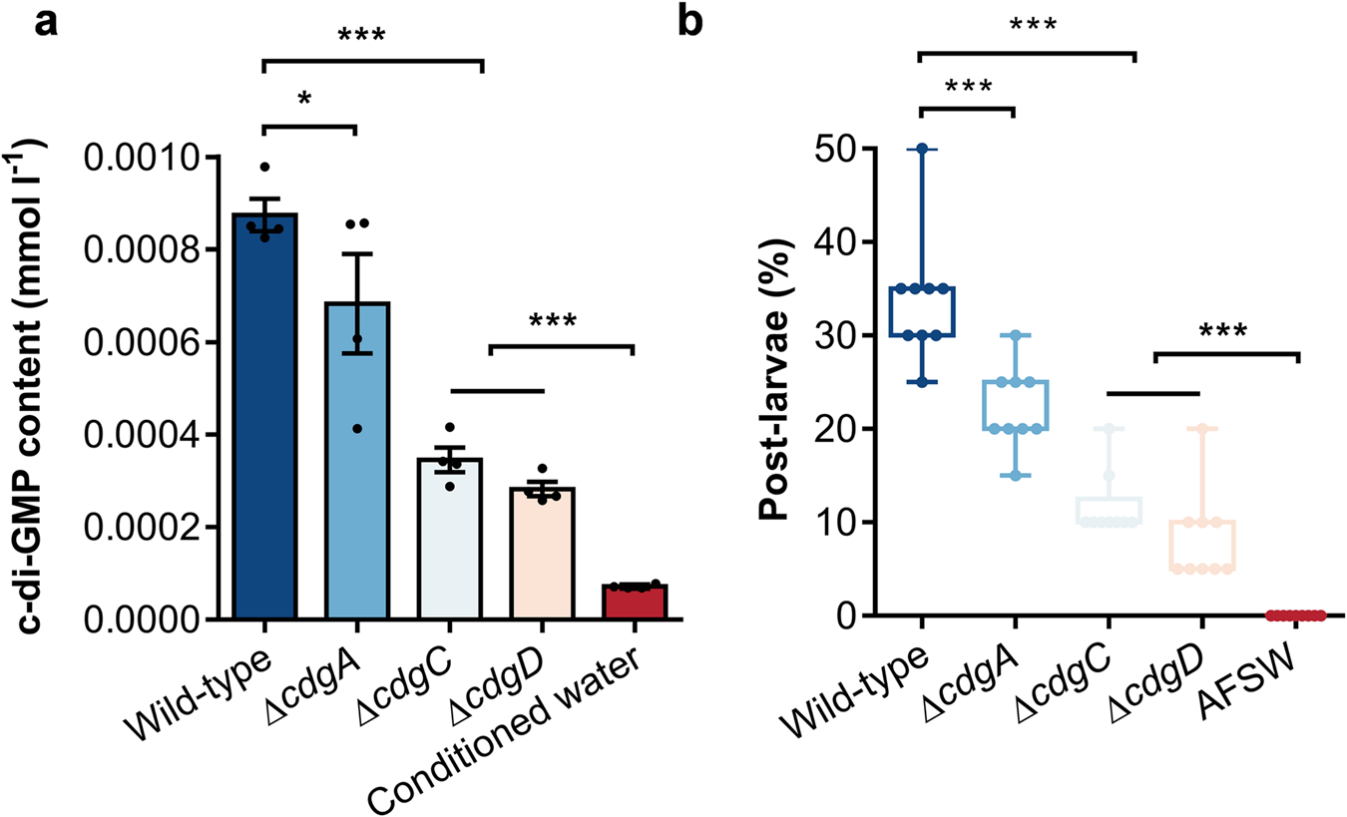
Effect of the wild-type strain and three mutant strains on c-di-GMP content, larval settlement and metamorphosis. **a** The c-di-GMP content of the wild-type and mutant biofilms. Boxes show means ± standard error of each treatment of 4 biological replicates. **b** The inducing activity of biofilms on larval settlement and metamorphosis. Box limits, upper and lower quartiles; show all points, 9 biological replicates. Each replicate group has 20 larvae. Statistical differences were determined by Wilcoxon test for each pair. The horizontal lines indicate no significant difference (*p* > 0.05); *p < 0.05; ***p < 0.001.

The genes containing GGDEF domains, which is responsible for c-di-GMP synthesis were screened from the genome of *P. marina*. Mutant strains with deletion of each of the three genes were successfully constructed, named *cdgA* (*orf01047*), *cdgC* (*orf01996*) and *cdgD* (*orf02758*) (Supplementary Fig. 1a). The c-di-GMP content of biofilms of mutant strains were significantly lower compared to wild-type strain, which was 8.7 × 10^−^^4^ mmol l^−^^1^ (Fig. 1a. *p* = 0.030). The c-di-GMP content of Δ*cdgA* biofilm was 6.8 × 10^−^^4^ mmol l^−^^1^. The c-di-GMP content in biofilms of Δ*cdgC* and Δ*cdgD* exhibited no significant difference, which were 3.5 × 10^−^^4^ mmol l^−^^1^ and 2.8 × 10^−^^4^ mmol l^−^^1^, respectively. In addition, we measured the content of c-di-GMP in the water used for culturing the wild-type biofilms. The c-di-GMP concentration of water was 7.2 × 10^−^^5^ mmol l^−^^1^.

The biofilms of Δ*cdgA*, Δ*cdgC*, and Δ*cdgD* showed lower inducing activity in contrast to the wild-type biofilms significantly (Fig. 1b, *p* = 0.001). The inductive effect of wild-type was about 33.89%, Δ*cdgA* and Δ*cdgC* biofilm induced to 22.22% and 11.67%, a reduction of 33.43% and 65.57%, respectively. Δ*cdgD* did not differ from the autoclaved filtered seawater (AFSW) group and had no inducing activity on larval metamorphosis. The results of the correlation analyses indicated that the inducing activity of both the wild-type and mutant strains showed high significant and positive relationship with the bacterial c-di-GMP content (*r* = 0.836, *p* < 0.001).

### Deletion of c-di-GMP synthesis genes reduced extracellular polysaccharide and protein content of biofilms

**Fig. 2:**
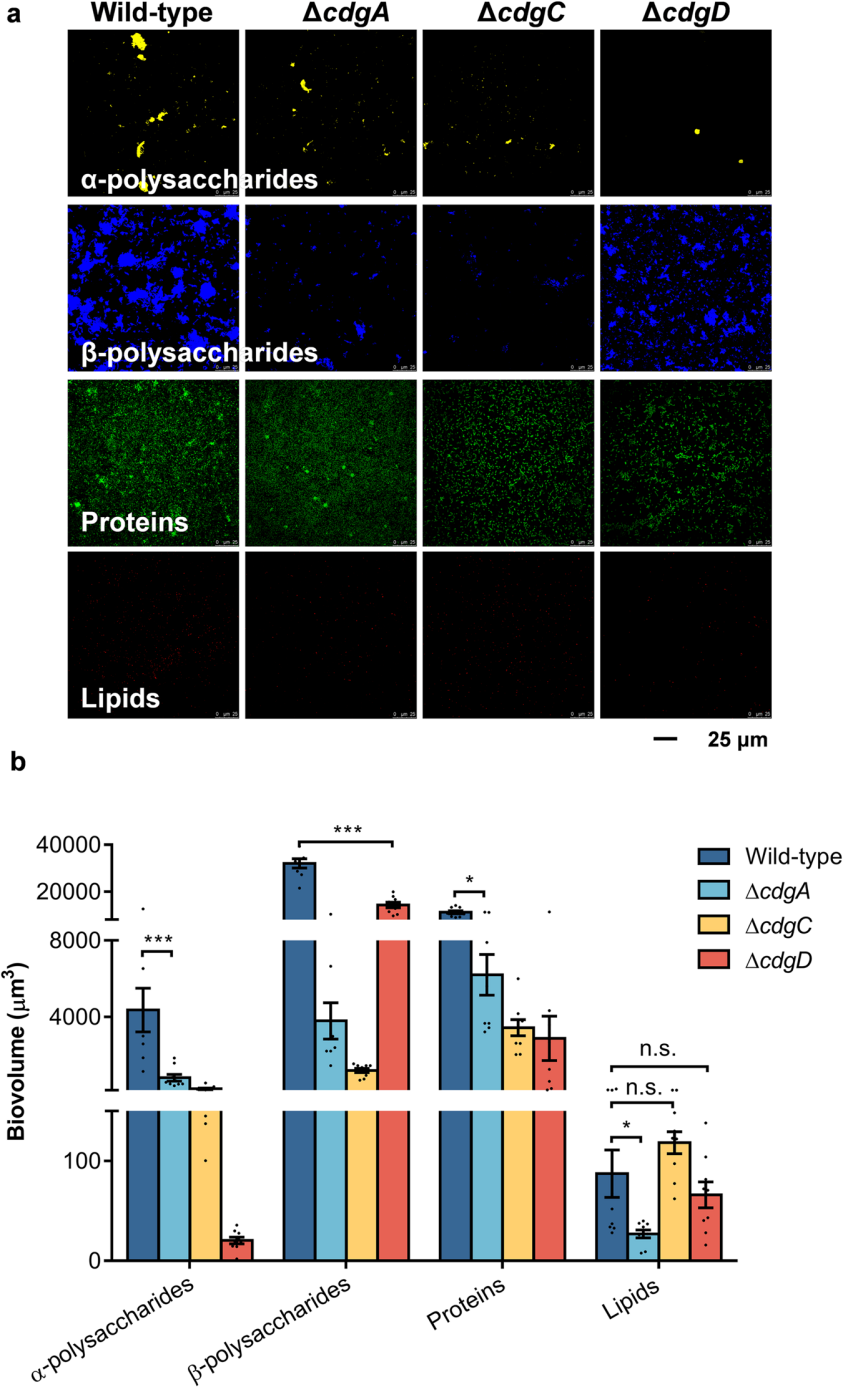
The effect of c-di-GMP synthesis genes deletion on the contents of α-polysaccharides, β-polysaccharides, proteins, and lipids in biofilms. **a** The CLSM images of biofilm EPS. **b** The biovolume analysis of biofilm EPS. Error bars represent standard errors of 9 biological replicates. Statistical differences were determined by Wilcoxon test for each pair. n.s., not significant (*p* > 0.05); *p < 0.05; ***p < 0.001.

In contrast to the wild-type strain, the three mutant strains exhibited significant reductions in the contents of extracellular α-polysaccharides, β-polysaccharides, and proteins (Fig. 2a). The contents of α-polysaccharides decreased by more than 80%, β-polysaccharides by more than 50%, and proteins by more than 40% (Fig. 2b). The extracellular lipid content of Δ*cdgA* strain exhibited a statistically significant decrease in comparison to the wild-type biofilms (*p* = 0.0217), while there was no significant change in Δ*cdgC* (*p* = 0.289), and Δ*cdgD* (*p* = 0.860) (Fig. 2b).

### C-di-GMP induced metamorphosis of *M. coruscus* larvae

**Fig. 3:**
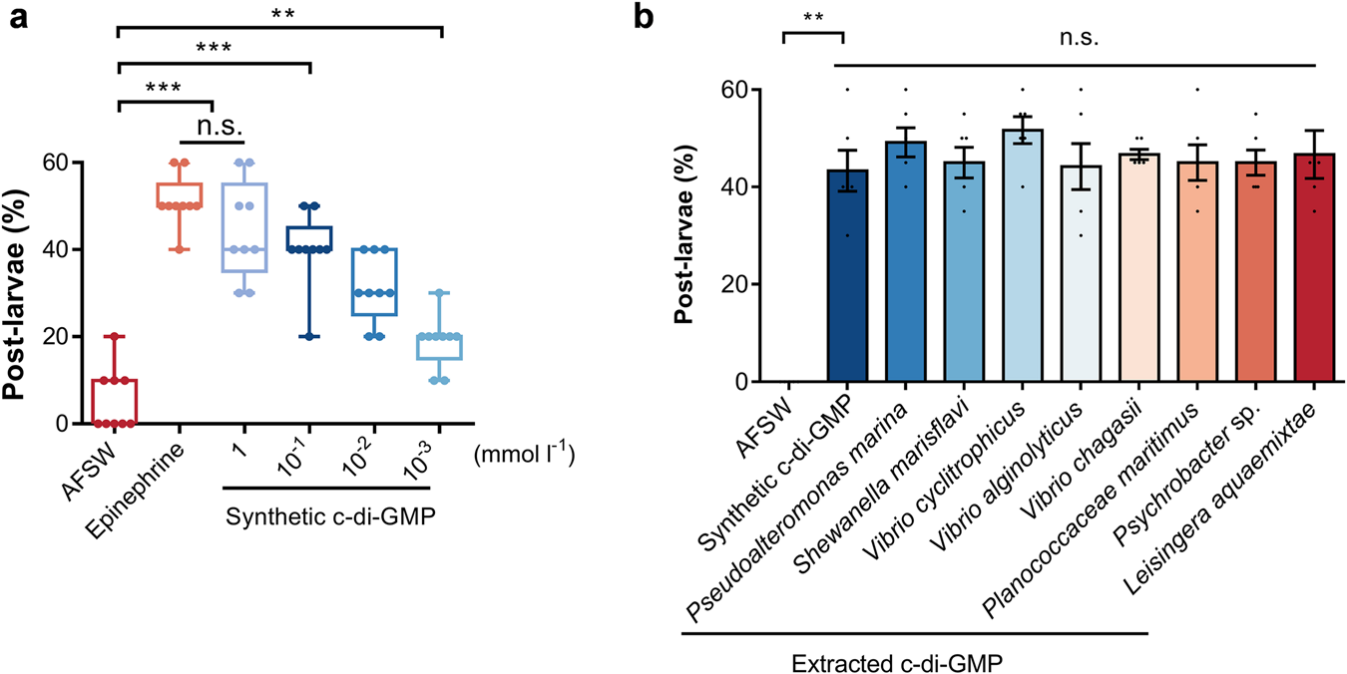
Effect of c-di-GMP on metamorphosis of *M. coruscus* larvae. **a** The inducing activity of synthetic c-di-GMP product on larval metamorphosis. Box limits, upper and lower quartiles; show all points, 9 biological replicates. **b** The inducing activity of extracted c-di-GMP of eight strains. Error bars represent standard errors of 6 biological replicates. Statistical differences were determined by Wilcoxon test for each pair. n.s., not significant (*p* > 0.05); **p < 0.01; ***p < 0.001.

c-di-GMP induced metamorphosis in a content-dependent manner (Fig. 3a). At a concentration of 1 mmol l^−^^1^, c-di-GMP solution showed the highest inducing activity of about 44.44%, which was comparable to that of epinephrine at the same concentration (Fig. 3a, *p* = 0.175). All four concentrations of c-di-GMP solution-treated groups had higher metamorphosis rates than blank group, with the lowest inducing activity was 18.89% at 10^−^^3^ mmol l^−^^1^ (Fig. 3a, *p* = 0.003). The inducing activity of natural c-di-GMP extracted from various marine bacteria was tested, and the results showed that c-di-GMP extracted from eight strains had the same inducing effect as the synthetic c-di-GMP at the same concentration (Fig. 3b). In addition, the tested marine bacteria showed different amount of c-di-GMP (Supplementary Fig. 2).

### Inhibition of the STING signaling pathway reduced the larval metamorphosis stimulated by c-di-GMP

**Fig. 4:**
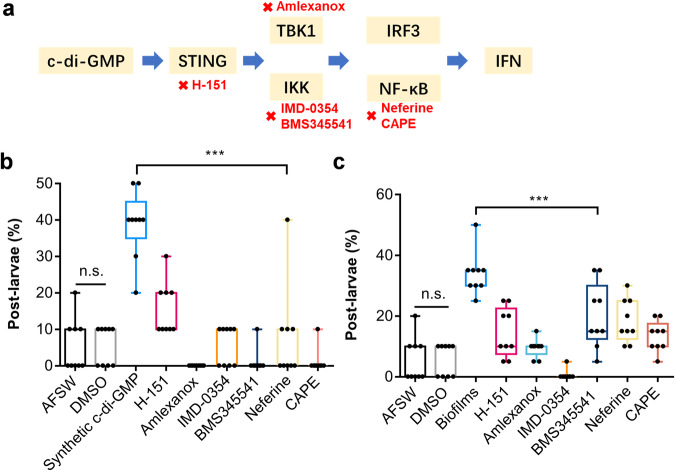
The inducing activity of c-di-GMP on treated larvae with inhibitors. **a** Targets of inhibitors in the c-di-GMP-STING signaling pathway. **b** The inducing activity of c-di-GMP on treated larvae with inhibitor. Synthetic c-di-GMP: 10^−1^ mmol l^−^^1^ synthetic c-di-GMP solution. DMSO: the solution of 10^−1^ mmol l^−^^1^ DMSO solution; the inhibitors were at concentration of 10^−1^ mmol l^−^^1^. The same as below. **c** The inducing activity of wild-type biofilms of *P. marina* on inhibitor-treated larvae. Box limits, upper and lower quartiles; show all points, 9 biological replicates. Statistical differences were determined by Wilcoxon test for each pair. n.s., not significant (*p* > 0.05); ***p < 0.001.

To identify potential receptors responding to c-di-GMP in *M. coruscus*, we conducted pharmacological experiments to test the effects of inhibitors on larval metamorphosis. All six inhibitors including H-151, Amlexanox, IMD-0354, BMS345541 hydrochloride (BMS345541), Neferine and Caffeic acid phenethyl ester (CAPE) reduced the inducing ability of c-di-GMP (*p* < 0.001) or wild-type biofilm on larval metamorphosis (*p* < 0.001) (Fig. 4b, c). The survival rate of larvae during the 96 h of the experiment was more than 80% (see Supplementary Fig. 3a).

### *McSTING* gene silence diminished larval metamorphosis

**Fig. 5:**
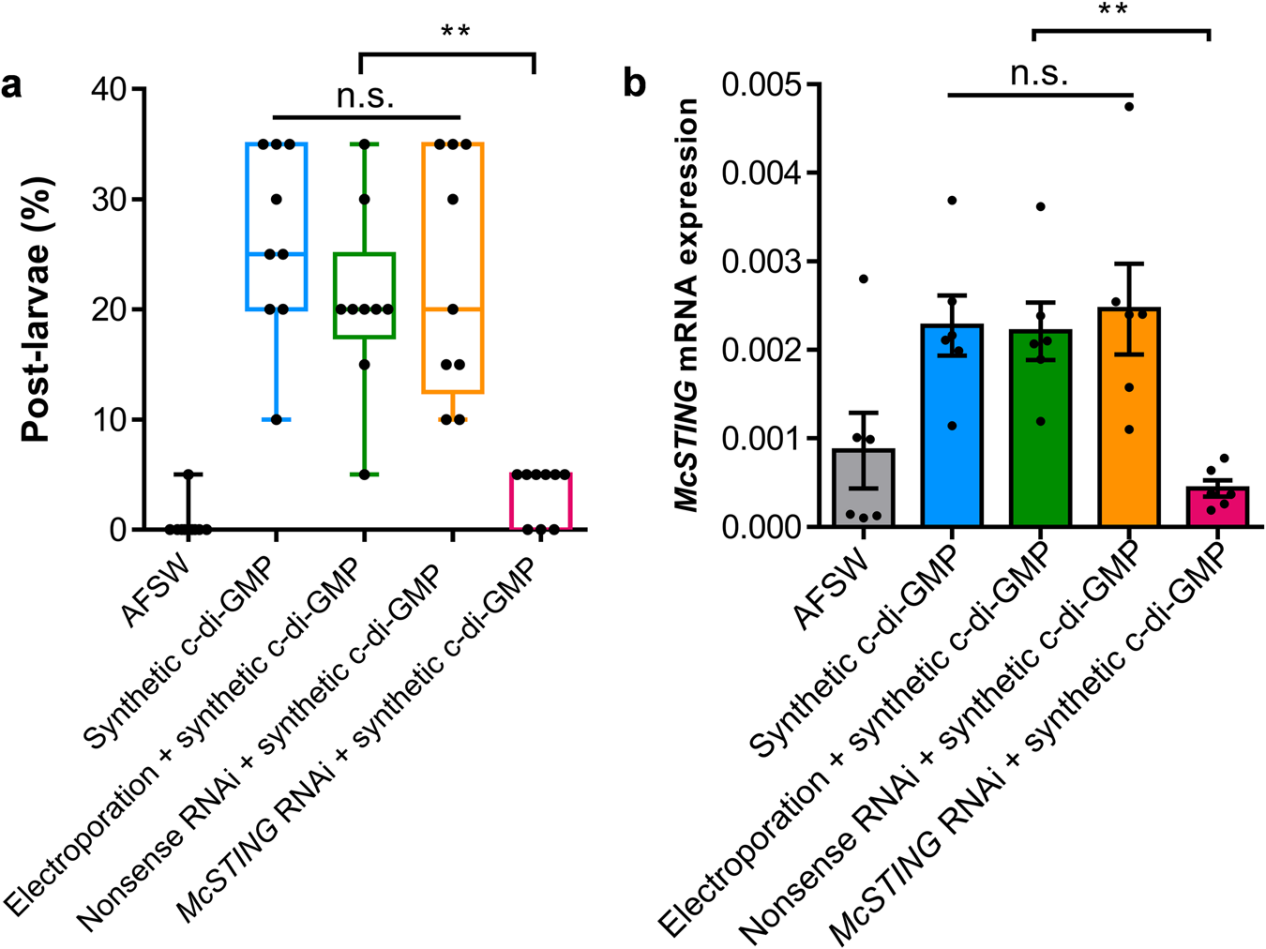
Effects of *McSTING* gene silencing on *McSTING* expression and larval metamorphosis. **a** Percentage of larvae metamorphosed at 48 h. Electroporation + synthetic c-di-GMP: Larvae were electroporated with nothing and then stimulated with synthetic c-di-GMP solution. Nonsense RNAi+synthetic c-di-GMP: Larvae were electroporated with 0.8 µmol l^−^^1^ nonsense siRNA and then stimulated with synthetic c-di-GMP solution. *McSTING* RNAi+synthetic c-di-GMP: Larvae were electroporated with 0.8 µmol l^−^^1^ *McSTING* siRNA and then stimulated with synthetic c-di-GMP solution. The same as below. Box limits, upper and lower quartiles; show all points, 9 biological replicates. **b** qRT-PCR expression of the *McSTING* in electroporated and control larvae. Error bars represent standard errors of 6 biological replicates. Statistical differences were determined by Wilcoxon test for each pair. n.s., not significant (*p* > 0.05); **p < 0.01.

To investigate the function of *Mc*STING during larval metamorphosis. Significant reduction in the rate of metamorphosis was apparent after gene silencing of *McSTING* (Fig. 5a, *p* < 0.001). After 10^−^^1^ mmol l^−^^1^ of c-di-GMP solution induction, the metamorphosis rate of untreated larvae was about 26.11%, whereas the metamorphosis rate of larvae electroporated by *McSTING* -siRNA decreased to 3.33% (Fig. 5a). In contrast, there were no significant changes in metamorphosis rate with electroporate only or electro-transfection with nonsense siRNA (Fig. 5a, *p* = 0.892). To test the effect of RNAi, samples of larvae after electroporation were collected for *McSTING* gene expression assay. The *McSTING* mRNA transcription of *McSTING* RNAi group comparatively inhibited compared to the electroporation group and nonsense RNAi group (Fig. 5b). Survival rates were shown in Supplementary Fig. 3b. We also found that *McSTING* expressed differently during larval metamorphosis, with the highest expression level in the pediveliger larval stage (Supplementary Fig. 4a).

### *Mc*STING protein binds to c-di-GMP

**Fig. 6:**
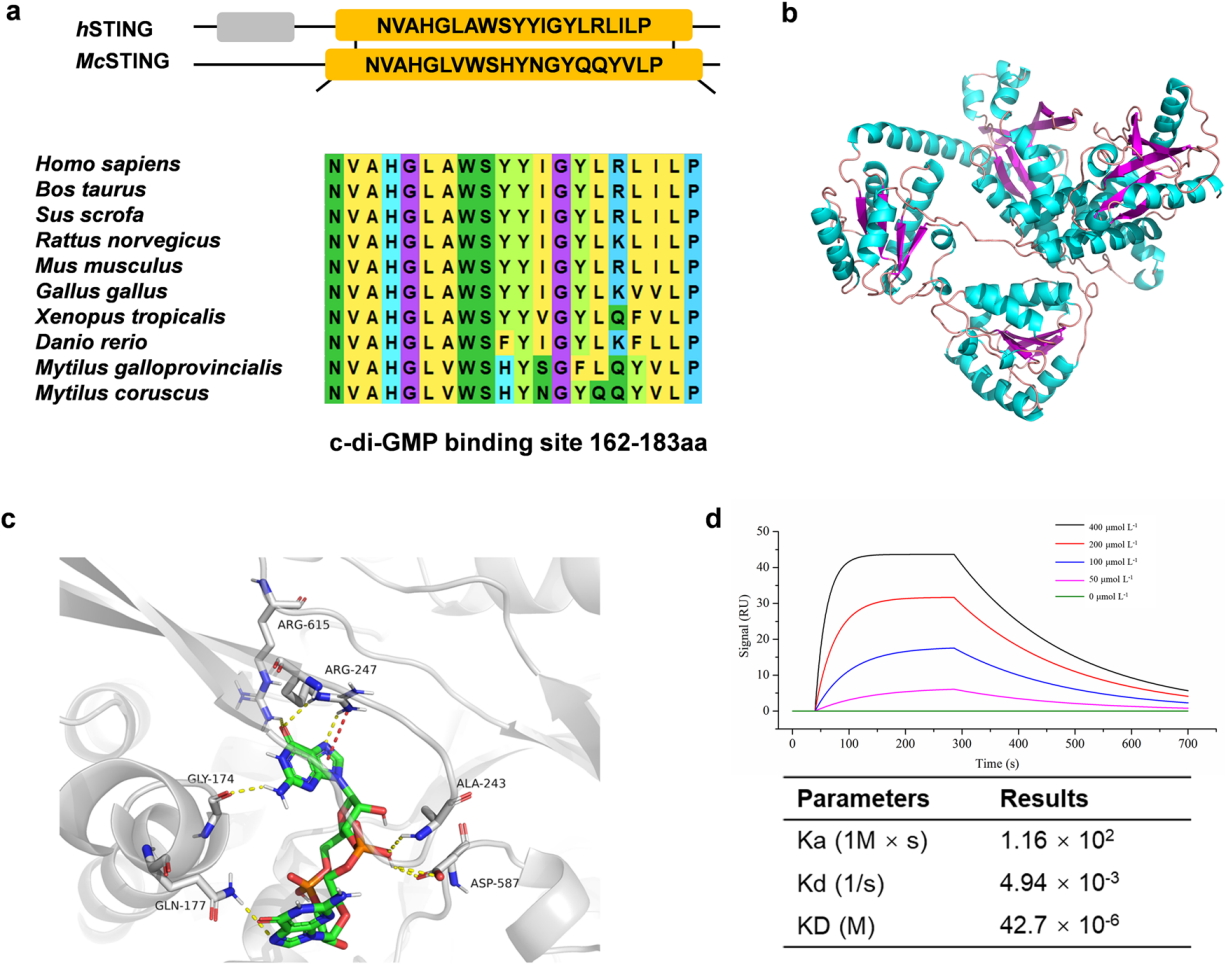
Binding assay between c-di-GMP and *Mc*STING. **a** Schematic of functional domains of human STING (*Hu*STING) and *Mc*STING, highlighting the evolutionary conservation of the c-di-GMP binding site. **b** Three-dimensional structure prediction of protein *Mc*STING. **c** The result of docking between *Mc*STING with c-di-GMP. The yellow dashed lines represent hydrogen bonds, and the red dashed lines represent π-cation interactions. **d** Purified *Mc*STING was tested for c-di-GMP binding by SPR. SPR sensor grams showing affinity measurements of an increasing range of *Mc*STING concentrations (0, 50, 100, 200 and 400 µmol l^−^^1^) at 298 K binding to c-di-GMP.

The full-length cDNA sequences of *McSTING* (MT123000.1) were obtained through rapid amplification of cDNA ends (RACE). We found a region labeled as c-di-GMP binding site within this sequence, and the sequence (from 162 to 183aa) is evolutionarily conserved (Fig. 6a). The three-dimensional structure of *Mc*STING was predicted (Fig. 6b). Figure 6c simulates the conformation with the lowest binding energy between c-di-GMP and *Mc*STING. It can be seen that there are binding sites between c-di-GMP and *Mc*STING (Fig. 6c). In detail, the NH in the amide bond of GLN177 forms a hydrogen bond with the nitrogen of position 3 (N3) of c-di-GMP indole. The carbonyl oxygen and hydroxyl oxygen of the side chain of ASP587 each form hydrogen bonds with the hydroxyl hydrogen of the phosphate. NH of ALA243 forms a hydrogen bond with the hydroxyl oxygen of the phosphate. In ARG247, the NH in the urea structure forms a hydrogen bond with the carbonyl oxygen, and NH_2_ forms a hydrogen bond with N3. Additionally, the N in NH_2_ interacts with the five-membered ring in the indole through a π-cation interaction. In ARG615, the NH_2_ in the urea structure forms a hydrogen bond with the carbonyl oxygen, and a hydrogen bond occurs between the carbonyl oxygen in GLY174 and the NH_2_ in the indole (Fig. 6c). The kinetics and affinity parameters are shown in Fig. 6d. Ka means association rate constant and Kd means dissociation rate constant and they were calculated by Trace Drawer software. KD is calculated by the equation: KD=Kd/Ka. It indicates the affinity unit or equilibrium constant^18^. There is concentration-dependent binding of the recombinant protein *Mc*STING to synthetic c-di-GMP with a KD of 42.7 × 10^−6^ M.

## Discussion

**Fig. 7:**
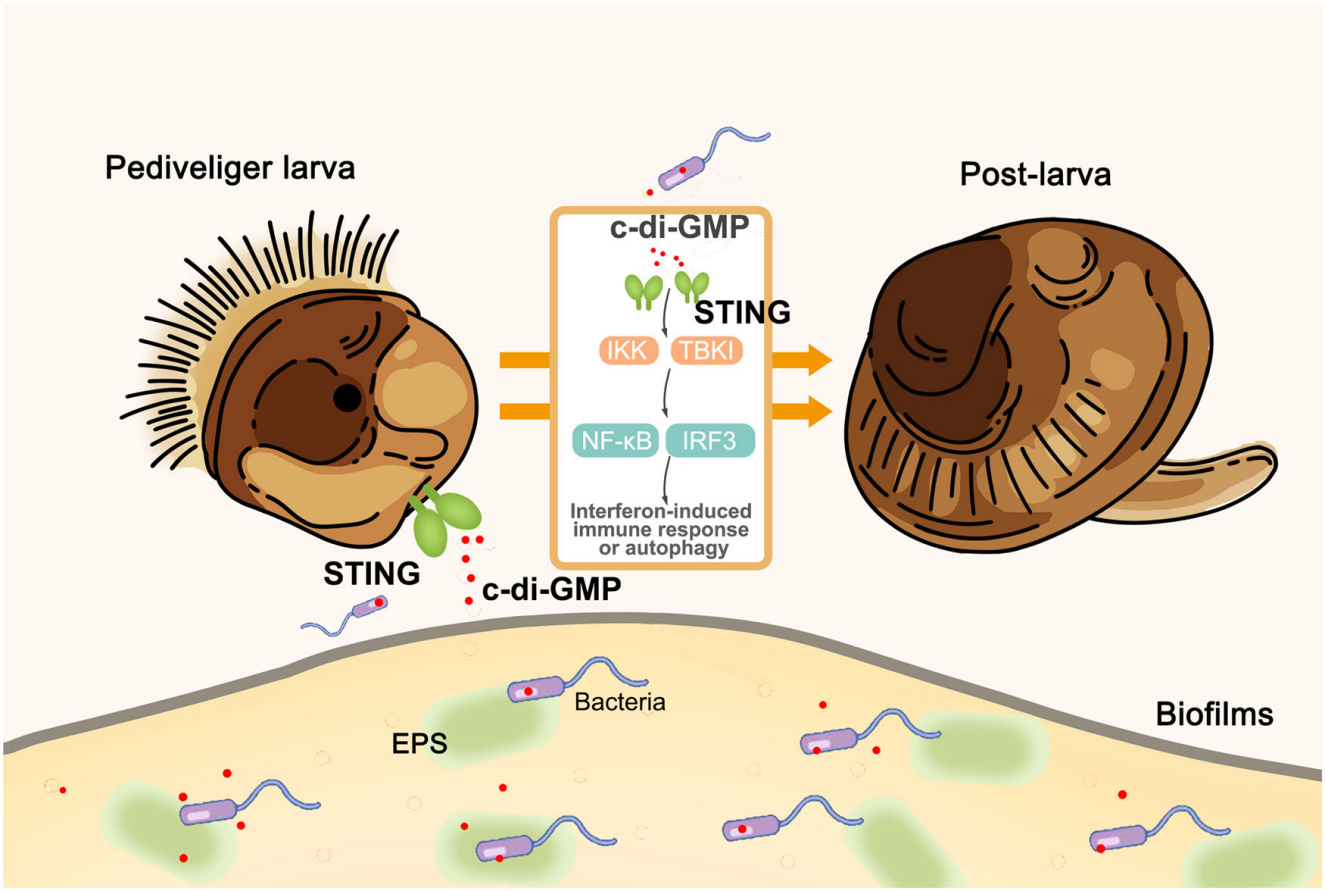
Bacterial c-di-GMP regulates larval settlement and metamorphosis through the STING signaling pathway in mussels *M. coruscus.* The “Pediveliger larva” and “Post-larva” elements referred to Hu et al. ^69^.

In previous studies, bacteria have been found to produce molecules that induce larval settlement and metamorphosis, such as histamine, a molecule from algae, which leads to the metamorphosis of sea urchins^19^. Arrays of phage tail-like structures, produced by *P. luteoviolacea*, that initiate metamorphosis of *Hydroides elegans*^20^. However, these molecules are usually produced by specific bacteria. There is a lack of universal bacterial molecules capable of inducing settlement and metamorphosis. Here, the results revealed that a ubiquitous bacterial molecule c-di-GMP triggers metamorphosis of mussel larvae through the STING receptor (Fig. 7).

*Pseudoalternomonas* is a widely distributed genus in marine environment can easily form biofilms and plays an important role in invertebrate-associated biological processes^21–23^. Huang and Hadfield using transposon mutagenesis developed two Hawaiian strain of *P. luteoviolacea* that lack the ability to trigger larval settlement and metamorphosis of *H. elegans* and they found that there are domains related to the mutant effect presence of a GGDEF peptide-encoding sequence responsible for synthesis of c-di-GMP^24^. C-di-GMP was first reported more than three decades ago as a second messenger in *Acetobacter xylinum*^25^. Now c-di-GMP had been discovered in almost all species of bacteria as a second messenger to coordinate biofilm formation, cell aggregation and bacterial motility^25–28^. In this study, we observed that the individual deletions of c-di-GMP synthesis genes in *Pseudoalternomonas marina* all resulted in a decrease in c-di-GMP levels. However, the extent of reduction varied. Pfam analysis (http://pfam-legacy.xfam.org/) showed that *cdgA* contains only GGDEF structural domain, *cdgC* contains GGDEF, EAL and GAF_2 structural domains, and *cdgD* contains GGDEF, EAL and dCache_3 structural domains (Supplementary Fig. 1b). The double Calcium channels and Chemotaxis receptors (dCache) domains are widely distributed extracellular sensor in bacteria and is found in almost all major types of prokaryotic transmembrane signal transduction proteins including c-di-GMP synthases^29^. cGMP phosphodiesterase/adenyl cyclase/FhlA (GAF) domains are very common small molecule binding domains that are typically participate in signal transmission processes as part of proteins^30^. Thus, the c-di-GMP concentrations and the inducible activities of bacteria in the biofilm were higher than those of Δ*cdgC* and Δ*cdgD* after the deletion of *cdgA*, which contains only the GGDEF structural domain. This could be attributed to the fact that the deletion of *cdgC* and *cdgD* not only decreased the concentrations of bacterial c-di-GMP but also affected the physiological functions of the bacteria. Studies in some bacteria suggest there is a regulation of c-di-GMP on EPS^31–34^. We therefore measured the EPS content of mutant bacteria, which showed a considerable decrease in extracellular polysaccharides and proteins. Given that the ability of EPS to induce larval metamorphosis has been widely demonstrated^35–38^. We initially speculated that the deletion of c-di-GMP biosynthesis genes led to a decrease in c-di-GMP content, which altered the inducing activity of larval metamorphosis by changing the EPS composition of the biofilm.

C-di-GMP can also affects important cellular activities in higher eukaryotes, including plant cellulose synthesis^39^, DNA synthesis in animal cells^40^, cell cycle^41^ and cell proliferation^42^. The c-di-GMP derived from the eight strains of marine bacteria tested for this study had the same ability to induce metamorphosis in mussel larvae as the commercial synthetic product. This study represents the evidence for the involvement of c-di-GMP in larval recruitment. Given that c-di-GMP is a ubiquitous signaling molecule in bacteria^43^, it has the potential to be a universal bacterial molecule that induces larval metamorphosis. The determination of c-di-GMP levels in the conditioned water raised another question. Under laboratory testing conditions, the most effective induction of larval metamorphosis occurred at concentrations of 1 mmol l^−^^1^ (or even higher) (Fig. 3a), while the c-di-GMP content in the biofilm and conditioned water was consistently below 10^−^^3^ mmol l^−^^1^ (Fig. 1a). Additionally, we confirmed the presence of c-di-GMP in the conditioned water during biofilm formation (Supplementary Fig. 5). These results suggest that while c-di-GMP can be released outside the bacteria during biofilm-induced larval settlement and metamorphosis, it did not reach a level that induced a similar effect. This implies the involvement of other substances in inducing larval attachment and metamorphosis. We propose that c-di-GMP, as a bacterial second messenger, may regulate larval metamorphosis both by direct regulation and by indirectly modulating other substances.

To elucidate the molecular mechanism of c-di-GMP-induced larval metamorphosis, we needed to turn at the larval side to find the receptor that responds to c-di-GMP. C-di-GMP has numerous binding proteins, although the majority of these proteins are found in bacteria^44–46^. Bacterial c-di-GMP were also found to bind with mouse STING and activated the innate immune signaling pathway^46,47^. In recent years, research on the function and origin of STING has gradually expanded. Studies in the marine invertebrate sea anemone *Nematostella vectensis* have revealed that autophagy induction is a primordial function of STING^48^. Even in the closest living relatives of animals, the choanoflagellate *Monosiga brevicollis*, STING has been found to respond to cyclic dinucleotides 2’3’ cGAMP and play a role in the immune response^49^. In the present study, we found that treatment of larvae with inhibitors of STING and downstream signaling molecules suppressed the inducing capability of c-di-GMP and *P. marina* biofilm on the metamorphosis. These findings suggest a potential involvement of the STING and downstream signaling molecules in larval metamorphosis.

Next, our focus shifts to understanding the function STING in larval metamorphosis. Due to the large variations in STING during evolution, the lack of studies on the structure and function of STING in invertebrates^50^, we need further verification to support the pharmacological results. We obtained the full-length cDNA sequence of the *McSTING*. In *Mc*STING, we identified a highly conserved c-di-GMP binding domain by comparing this region with the amino acid sequences of STING in other animals. Then the crucial role of *Mc*STING in larval metamorphosis was confirmed by RNA interference (RNAi). The similarity in metamorphosis rates between the electroporation + c-di-GMP group and the c-di-GMP group suggests that the electroporation did not affect larval metamorphic abilities. Furthermore, transfection of nonsense siRNA did not alter the inducibility of c-di-GMP on larval metamorphosis or the expression level of the *McSTING* gene. This indicates that the reduced metamorphosis rate in the *McSTING* RNAi group is attributed to the suppression of *McSTING* expression, thereby affecting larval metamorphic abilities. The results of *McSTING* gene expression confirmed the involvement of *McSTING* in metamorphosis. The expression level of *McSTING* is highest during the pediveliger stage, which marks the onset of metamorphosis, suggesting that *McSTING* is involved in this process (Supplementary Fig. 4a). As we known that STING is an innate immune molecular^51^, we tested the *McSTING* gene expression in different tissues of mussels. The high gene expression of the *McSTING* in the tissues exposed to the external environment, such as mantle and gill, also underscore its effect on immunity (Supplementary Fig. 4b). Therefore, we proposed that c-di-GMP induces metamorphosis in larvae via the innate immune pathway. A study suggests that innate immune signaling likely plays a role in various processes during metamorphosis in an ascidian species, *Boltenia villosa* including the absorption of larval tissues, migration of mesenchyme cells and tunic maturation or adhesion that occurs during larval metamorphosis^52^. Additionally, innate immune signaling during the larval stage may be responsible for the detection of bacterial cues for settlement by ascidian larvae^53^. During the metamorphosis in marine invertebrates from planktonic to benthic life, the habitat undergoes significant changes, requiring the adaptation of interacting systems such as the immune system to these alterations in the surrounding environment^53–56^. This is reinforced by the results of this study, linking the metamorphosis of mollusc larvae to innate immunity.

Finally, we recombined and purified the *Mc*STING protein and performed an interaction analysis with c-di-GMP. In this study, we attempted to detect the binding of the recombinant protein *Mc*STING to bacterial c-di-GMP using Surface Plasmon Resonance (SPR) technique. The affinity constant between c-di-GMP and *Mc*STING was 42.7 × 10^−^^6^ M, a value within the range typically associated with the binding of small molecules to proteins, indicating that *Mc*STING can combine with c-di-GMP^57^. The KD value indicating that the binding ability of c-di-GMP to STING is strong when the concentration reaches 42.7 × 10^−^^3^ mmol l^−^^1^. This result corresponds to the physiological concentration of c-di-GMP, which is sufficient to induce larval metamorphosis (higher than 42.7 × 10^−^^3^ mmol l^−^^1^). However, human STING (hSTING) binds c-di-GMP with micromolar affinity in KD value^47^, lower compared to that of *M. coruscus*. Due to differences in amino acid residues and the number of hydrogen bonds at the interaction site between STINGs and c-di-GMP, they exhibit different binding affinities, resulting in different KD values.

Our research revealed a new role of bacterial c-di-GMP that triggers mollusc larval metamorphosis transition and definitively established the crucial role of STING in responding to the metamorphosis processes mediated by c-di-GMP in invertebrate. This discovery holds dual significance. Firstly, it unveils the highly conserved nature of the c-di-GMP binding domain in STING from marine invertebrates to humans, implying an ancient mechanism involved in c-di-GMP interaction with bacteria. Secondly, the identification of a new function for STING underscores its pivotal role in animal development. This work extends knowledge in the interaction of bacteria and host development in marine ecosystems.

## Methods

### Construction of a strain with deletion of c-di-GMP synthesis gene

The strains, plasmids and primers were detailed in Table S1. Three genes containing c-di-GMP synthesis domains were selected from the genome of *P. marina* ECSMB14103^17^ and named *cdgA*, *cdgC*, and *cdgD*. The constructions of c-di-GMP synthesis gene deletion mutant strains Δ*cdgA*, Δ*cdgC*, and Δ*cdgD* were based on an approach of homologous recombination^58^. PCR products were ligated to pK18 after enzymatic digestion and then transformed to *E. coli* WM3064. Conjugation and transformation to wild-type strain referring to Zeng et al. ^59^. The wild-type and mutants of *P. marina* were cultured in 2216E medium (0.5% peptone, 0.001% ferric citrate and 0.1% yeast extract) at pH 7.6–7.8, 25 °C.

### Experimental organisms

Adult mussel *M. coruscus* were sampled from Zhoushan Islands, Zhejiang Province (122°77’E, 30°73’N) of China. In this study, spawning and larval culture were conducted with reference of Yang’s method^60^. Samples were collected from the trochophore developmental stages of mussel larvae. *Isochrysis zhangjiangensis* were used as bait for larvae. The larvae are used for metamorphosis experiments as they develop into pediveliger larvae. Pharmacology experiments are conducted in 6-well plates (15.7 × 17.2 mm), while biofilm experiments are conducted in glass culture dishes (ø64 × 19 mm). They were kept at 18 °C in darkness for 48 h, and the behavior and body structure of the larvae were observed with stereo microscopes. Settlement and metamorphosis of mussel larvae includes stopping swimming, shedding velum, acquisition of gills and shell growth. In this test, percentage of post-larvae indicates the inducing activity. The mortality of larvae was measured 96 h after each test. Each experiment was performed 6 replicates.

### Biofilm formation and composition analysis

The procedures for biofilm formation followed the previous method of Yang et al. ^61^. The strains were incubated in glass flasks containing Zobell 2216E medium over 12 h. The medium was discarded, and the bacteria were collected after centrifugation, then suspended in AFSW by mixing. This process was repeated 3 times to wash the bacteria and remove the medium. Finally, the washed bacteria were suspended in AFSW to prepare bacterial stock solution for counting bacterial density, and the stock solution was diluted to 1 × 10^8^ cells ml^−^^1^ with AFSW for the preparation of biofilms. The sterilized glass slides (2.5 cm × 3.8 cm) and 20 ml of diluted bacterial solution were added to each petri dish, and after incubation at 18 °C for 48 h, the slides were gently washed in AFSW to remove the unattached bacteria The biofilm was obtained for subsequent experiments.

The content of extracellular substance on the biofilm was observed and quantified referring to Liang et al. ^62^. Briefly, the biofilm was stained using dyes listed in Table S2 and protected from light. Images of the stained biofilm were obtained by Confocal Laser Scanning Microscopy (CLSM) (Leica SP8, German), and quantitative analysis was conducted using Image J 1.52a (USA). For each strain and substance, three slides were prepared, with three fields of view captured per slide, resulting in a total of nine replicates.

### Extraction and quantification of c-di-GMP

The experiment was carried out referring to the protocol of Roy et al. ^63^. and Peng et al. ^13^. Eight marine bacterial strains were isolated and purified from the natural biofilm in the East China Sea (Table S3). Bacterial solution was centrifuged to discard the supernatant, and the bacterial pellet was retained. When the pellet was dried, c-di-GMP extraction solution (0.1% formic acid, 40% acetonitrile, 40% methanol, and 19.9% water by volume) was added. The pellet was then vortexed to mix with the extraction solution. Subsequently, the mixture was incubated on ice and centrifuged, allowing c-di-GMP to be extracted into the supernatant. Part of the supernatant was subjected to quantification using LC-MS/MS (Waters, USA). The remaining supernatant was separated and purified by high performance liquid chromatography, rotary evaporation, and freeze-drying to obtain c-di-GMP solid referred to Rao et al. ^64^. Equal masses of c-di-GMP solid were dissolved in AFSW to prepare a 0.01 mmol l^−^^1^ test solution for the subsequent larval metamorphosis experiments.

### Pharmacological treatments

The commercial synthetic and extracted c-di-GMP were standardized to a concentration of 0.1 mmol l^−^^1^ as the good inducing effect on larval metamorphosis. Subsequently, their inducible potential for larval metamorphosis was assessed. The chemicals used in this study included c-di-GMP, H-151, IMD-0354, Amlexanox, Neferine, CAPE, and BMS345541. The targets of each pharmaceutical on the c-di-GMP downstream signaling pathway were shown in Fig. 4a and Table 1. The selective and covalent antagonist of STING H-151 was examined. The inhibitors of TBK1 and IKK include Amlexanox, IMD-0354 and BMS345541 hydrochloride were used. The inhibitors of NF-κB include Neferine and Caffeic acid phenethyl ester were investigated. Firstly, the chemicals were dissolved in distilled water or dimethyl sulfoxide (DMSO) and subsequently diluted with AFSW to prepare stock solutions. The stock solutions were further diluted with AFSW to obtain the desired concentrations for testing. All test solutions were prepared on the day of the experiment to minimize storage time.

**Table 1:** Pharmacological compounds tested, inhibiting activity, target and bioassay concentration

Name	Activity	Target	Manufacture and Cat. No.	Concentration (mmol l^-1^)	
				Stock solution	Test solution
c-di-GMP	regulates biofilm formation, motility, and virulence	STING	HY-107780	1	1,10^−^^1^, 10^−^^2^,10^−^^3^
H-151	a selective and covalent antagonist of STING	STING	HY-112693	10^−^^1^	10^−^^1^,10^−^^2^,10^−^^3^
Amlexanox	a specific inhibitor of IKKε and TBK1	TBK1	HY-B0713	10^−^^1^	10^−^^1^,10^−^^2^,10^−^^3^
IMD-0354	a selective IKKβ inhibitor which inhibits NF-κB activity	IKK	HY-10172	10^-1^	10^−^^1^,10^−^^2^,10^−^^3^
BMS345541 hydrochloride	a IKKα/IKKβ inhibitor	IKK	HY-10518	10^−^^1^	10^−^^1^,10^−^^2^,10^−^^3^
Neferine	a major bisbenzylisoquinline alkaloid can strongly inhibits NF-κB activation.	NF-κB	HY-N0441	10^−^^1^	10^−^^1^,10^−^^2^,10^−^^3^
Caffeic acid phenethyl ester	a NF-κB inhibitor	NF-κB	HY-N0274	10^−^^1^	10^−^^1^,10^−^^2^,10^−^^3^

In the larval pharmacological experiments, AFSW and a 10^−^^1^ mmol l^−^^1^ DMSO solution were set as negative controls, while a 10^−^^1^ mmol l^−^^1^ epinephrine solution served as a positive control. After a 2-hour exposure to the certain concentration of inhibitors in AFSW solution, the larvae were rinsed three times with AFSW and then transferred to 6-well plates containing biofilms or c-di-GMP solution. The effects of the inhibitors on biofilms and c-di-GMP-inducing activity of larval metamorphosis were tested. After a 2 h exposure to DMSO and epinephrine solution, the larvae were rinsed three times with AFSW and transferred to a new set of 6-well plates for the detection of inducing activity in the negative and positive controls.

### Sequence and qRT-PCR analysis of gene *McSTING*

We identified *STING* gene from the mussel *M. coruscus* transcriptome genes that are homologous to vertebrates *STING* and the primers were designed based on the partial *STING* sequences. Total RNA was extracted from the various tissues (foot, gill, hemolymph, adductor, digestive gland, mantle, labial palp, gut, and gonad) and development stages (trochophore, D larva, umbo, pediveliger, and post-larva) of *M. coruscus* using RNAiso Plus kit according to the instructions (Takara, Japan) and reversed transcription to cDNA (PrimeScript™ RT reagent Kit, Takara, Japan). Using RACE technique to obtain the full-length *McSTING*. The specific primers are shown in Table S4.

The sequence homology of the *McSTING* cDNA with other *STING*s was established using NCBI BLAST. Using open reading frame (ORF) Finder to predicted the ORF. The signature domains of the deduced proteins were identified using SMART Tool. The amino acid sequences were performed multiple sequence alignment using MEGA 11 (New Zeeland).

Based on the obtained sequence of *McSTING* gene, we designed primers for qRT-PCR based on the available sequences (Table S4). *α-Tublin* and *elongation factor 1α* (*EF-1α*) genes were used as reference gene. The relative mRNA transcript levels of *McSTING* were determined using an absolute quantification approach, as described by Li et al. ^65^. In brief, a standard template was prepared to construct a standard curve. Reaction mixtures comprising cDNA template, primers, FastStart Essential DNA Green Master (Roche), and sterile MilliQ water were assayed using a LightCycler 960 (Roche) instrument following qRT-PCR amplification protocols. Each group consisted of 6 replicates. The copy number of the target genes was calculated based on the Ct value using the standard curve.

### RNA interferon of *McSTING*

The *McSTING* and nonsense siRNAs were designed against the cDNA sequence of *McSTING* (Table S4). The *McSTING* siRNA can match with specific sequence within the ORF of the *McSTING* gene, while the nonsense siRNA does not match with any segment of the *McSTING* sequence, thus serving as a negative siRNA control. The siRNA was synthesized by GenePharma (Shanghai). Transfection by electroporation used in this study refer to the method of Barreto et al. ^66^. First, 1 ml of AFSW with target and nonsense siRNA were respectively mixed into a 1.5 ml tube and about 300 active pediveliger larvae of the same size were collected into them, then transferred the mixture to an electroporation cuvette. The sample was electroporated to transfect the target or nonsense siRNA into the larvae in a Gene Pulser Xcell (Bio-Rad, USA). The electroporation protocol was conducted followed as Liang et al., with the following parameters: A single pulse of 100 V for 5 ms, 10 pulses of 50 V administered for 20 ms, 1 s intervals between the pulses^67^. For the control group, the same electroporation procedure was taken but without any siRNA. After electroporation, the larvae were transferred to petri dish, and part of them was used for RNA extraction and expression analysis of *McSTING* gene, the others were used for the metamorphosis and survival bioassays. The metamorphosis bioassays were taken by adding the electroporated larvae to 10^−^^2^ mmol l^−^^1^ c-di-GMP solution. Twenty larvae per petri dish were placed in dark conditions at 18 ± 1 °C and record the rate of *M. coruscus* post-larvae. Larvae were collected for qRT-PCR analysis of *McSTING* after 48 h exposure. To study the effect of electro transfection on the survival rate of larvae, some larvae were placed in darkness at 18 ± 1 °C, fed with *Isochrysis zhanjiangensis* daily after electroporation and recorded the survival rate.

### *Mc*STING protein recombination and purification

When performing protein recombination and purification, we constructed a recombinant expression vector (3665-F2-1-pColdIII-JT, 1-716aa) for the full-length amino acid sequence of *Mc*STING. The recombinant plasmid DNA was transformed into competent cells of the expression host for induction in a 300 ml shaker flask at 37 °C. The induced culture was analyzed using SDS-PAGE to confirm the presence of the correctly sized protein bands. Subsequently, protein extraction and purification were carried out. Finally, the recombinant protein was identified through electrophoresis, obtaining gel bands and spots. A series of optimizations were performed using in-gel enzymatic digestion, and ultimately, LC-MS/MS technology was employed to obtain information on protein fragmentation, charge, peak profiles, and other relevant details for comparative analysis to determine the recombinant proteins.

### Determination of c-di-GMP-*Mc*STING protein interaction

A three-dimensional structure model of the protein was generated using the RoseTTAFold method in Rebetta, relying on the amino acid sequence. The quality of the *Mc*STING protein structure model was evaluated using Ramachandran plots. The modeled protein structure was then imported into MOE2015 software for structure preparation and energy minimization. The protein structure was designated as the receptor, and the structure of c-di-GMP was selected as the ligand. The docking method used was inducefit, only the conformation with the lowest binding free energy was chosen for analysis. The results were visualized and analyzed using PyMOL.

The determination of c-di-GMP-*Mc*STING protein interaction was analysis by SPR technique. First, we constructed full-length *McSTING* gene expression vector *Mc*STING-pColdIII (1-716aa) and transformed into receptor cells DH5α to extract plasmids. Then we transformed plasmids to *E. coli* Rossatta. The identified positive colonies were selected for expansion, the *Mc*STING protein was induced to be expressed. Finally, we extracted and purified the *Mc*STING protein. C-di-GMP purchased from MCE Company (China). The interaction between analyte c-di-GMP and ligand *Mc*STING protein were assayed by SPR technique at room temperature (298 K) on OpenSPR instruments (Nicoya, Canada)^68^. First, COOH sensor chip was inserted into the instrument. After cleaning the chip surface, we sampled and operated 200 µl *Mc*STING protein and blocking solution at 20 µl/min flow rate for 4 min separately. After each sample we used PBS buffer and air rinsed the flow channels. Observing baseline for 5 min to ensure stability. Finally, c-di-GMP solutions of varying concentrations were injected into SPR device to assay the interaction with *Mc*STING.

### Data analysis

In this experiment, data analysis was conducted using JMP software (version 10), and the Wilcoxon method was employed for non-parametric comparisons of the data.

### Reporting summary

Further information on research design is available in the Nature Research Reporting Summary linked to this article.

### Supplementary information

supplemental material

## Data Availability

The data analyzed in this study are available within the article and its supplementary files or from the corresponding author upon request.
