# Supplementary material for: Bacterial c-di-GMP triggers metamorphosis of mussel larvae through a STING receptor
Source: NPJ Biofilms Microbiomes. 2024 Jun 20;10:51. doi: 10.1038/s41522-024-00523-7 (PMC11190208; doi:10.1038/s41522-024-00523-7)
Supplement: Supplementary file 1 — supplemental material [file 41522_2024_523_MOESM1_ESM.pdf]

Supplementary Materials

Supplementary Table 1. Strains, plasmids and primers for deletion of *cdgA*, *cdgC*, and *cdgD* used in this study

**Strains or plasmids**	**Relevant characteristics**	**Source**
**Strains**		
P. marina ECSMB14103	Wild-type	Lab Stock
E. coli WM3064	RP4(tra) in chromosome, DAP^-	Lab Stock
Δ*cdgA*	In-frame deletion of *cdgA*	This study
Δ*cdgC*	In-frame deletion of *cdgC*	This study
Δ*cdgD*	In-frame deletion of *cdgD*	This study
Plasmids		
pK18mobsacB-ery	pK18mobsacB containing the erythromycin resistant gene from pHT304, Kan^r, Ery^r	Lab Stock
pK18mobsacB-ery-*cdgA*	Recombinant plasmid for deleting *cdgA*genes	This study
pK19mobsacB-ery-*cdgC*	Recombinant plasmid for deleting *cdgC*genes	This study
pK20mobsacB-ery-*cdgD*	Recombinant plasmid for deleting *cdgD*genes	This study
**Primers**		
cdgA-up-F	CGCGGATCCAACGACCAAATAGAAACG	
cdgA-up-R	CCGCTCGAGTTATAAGGCTTTTTAAAAG	
cdgA-down-F	CCGCTCGAGTAAATTCTCCCCCGAGAGTG	
cdgA-down-R	ACGCGTCGACATGCCTACGGTAACAAAA	
cdgA-L-F	CACCGCAAAGTGTTAT	
cdgA-L-R	AGTCCTGCTTTCGTTC	
cdgA-S-F	AAGCAATAGGGCAAGG	
cdgA-S-R	CCGCAGCGACAAGTAA	
cdgC-up-F	CGCGGATCCAGTGCTTGCTGGTGAATA	
cdgC-up-R	CCGCTCGAGAATTTATTATTCTTTTTTA	
cdgC-down-F	CCGCTCGAGCCCCACATCTGTAAAGTTAA	
cdgC-down-R	ACGCGTCGACGTCTGGAATTGCGTCATA	
cdgC-L-F	TGCAGGCACAGTCCAA	
cdgC-L-R	CGGAATAGGGTAAGAAATAA	
cdgC-S-F	ACATGCCAATCGTCAG	
cdgC-S-R	CCAATAACAAACCCAAA	
cdgD-up-F	CGCGGATCCCCGAGTAGTCCCAGTTCA	
cdgD-up-R	CCGCTCGAGGTATTTATTTGTCAGTGTTT	
cdgD-down-F	CCGCTCGAGTACCAGCCTTAAATTTGCTA	
cdgD-down-R	ACGCGTCGACCCGCCAGTAGACGATGTA	
cdgD-L-F	AAATAGTGAAATACCTGCTC	
cdgD-L-R	TTACGCCATCAACCAA	
cdgD-S-F	TGGCACGTCAAACTCT	
cdgD-S-R	ACCAGCACCAAGAAAC	

Supplementary Table 2. Dye used for confocal laser scanning microscopy and target

Dye	Target	Concentration	Detection wavelength
Concanavalin A, tetramethylrhodamine conjugate (ConA-TMR)	α-polysaccharide	944.8 μg ml^{-1}	552-578 nm
Calcofluor white M2R (CFW)	β-polysaccharide	189 μg ml^{-1}	254-432 nm
DiIC18(5) oil, 1,1’-dioctadecyl-3,3,3’,3’-tetramethylindodicarb-ocyanine perchlorate (DiD’oil)	Lipid	7.94 μg ml^{-1}	648-670 nm
Fluorescein isothiocyanate isomer I (FITC)	Protein	46.6 μg ml^{-1}	495-519 nm

Supplementary Table 3. The information of the eight marine bacteria

Solate	Closest match name of BLAST	Accession no. of closest match	Accession no	Similarity	Source
ECSMB14103	*Pseudoalteromonas marina*	MN746206	OQ931036	99.65%	Natural biofilm
ECSMB14101	*Shewanella marisflavi*	OP209749	OQ931038	99.72%	Natural biofilm
ECSMB14105	*Vibrio cyclitrophicus*	MN945399	OQ931037	99.86%	Natural biofilm
ECSMB14115	*Vibrio alginolyticus*	MN938185	OQ931040	99.72%	Natural biofilm
ECSMB14107	*Vibrio chagasii*	MN938232	OQ931041	94.29%	Natural biofilm
ECSMB14108	*Planococcus maritimus*	MK332598	OQ931042	99.59%	Natural biofilm
ECSMB13125	*Psychrobacter* sp.	MF537114	OQ931043	98.67%	Natural biofilm
ECSMC17901	*Leisingera aquaemixtae*	OQ324740	OQ931237	97.93%	*Mytilus coruscus*

Supplementary Table 4. Sequences of primers used for RACE amplification, qRT-PCR and RNAi of *McSTING* gene

Primer name	Sequence	Usage
McSTING-F	TTTCCATCCCTGAATCTC	PCR
McSTING -R	CTAAACCGTATGCCACAT	PCR
McSTING -5'-675	TCTCCTTCAGCACAGGCAA	5'RACE
McSTING -5'-495	GGAGAACTGCTATTCGTGGA	5'RACE
McSTING -3'-1908	GTATTTCCTTTTGGACCAGGCAGG	3'RACE
McSTING -3'-2001	GGACAGTATGCAGCACCAGTGGC	3'RACE
McSTING -RT-F1	ACAGTGTTACGGATGGCATTGAGAC	qRT-PCR
McSTING -RT-R1	TGCTGTTGTGTAGCCGCCTTCT	qRT-PCR
αTublin-F	TTGCAACCATCAAGACCAAG	qRT-PCR
αTublin-R	TGCAGACGGCTCTCTGT	qRT-PCR
EF-1α-F	CACCACGAGTCTCTCCCTGA	qRT-PCR
EF-1α-R	GCTGTCACCACAGACCATTCC	qRT-PCR
McSTING -siRNA	GUGGCAUGCUUAGAAGAAATT	RNAi
Nonsense -siRNA	UUCUCCGAACGUGUCACGUTT	RNAi

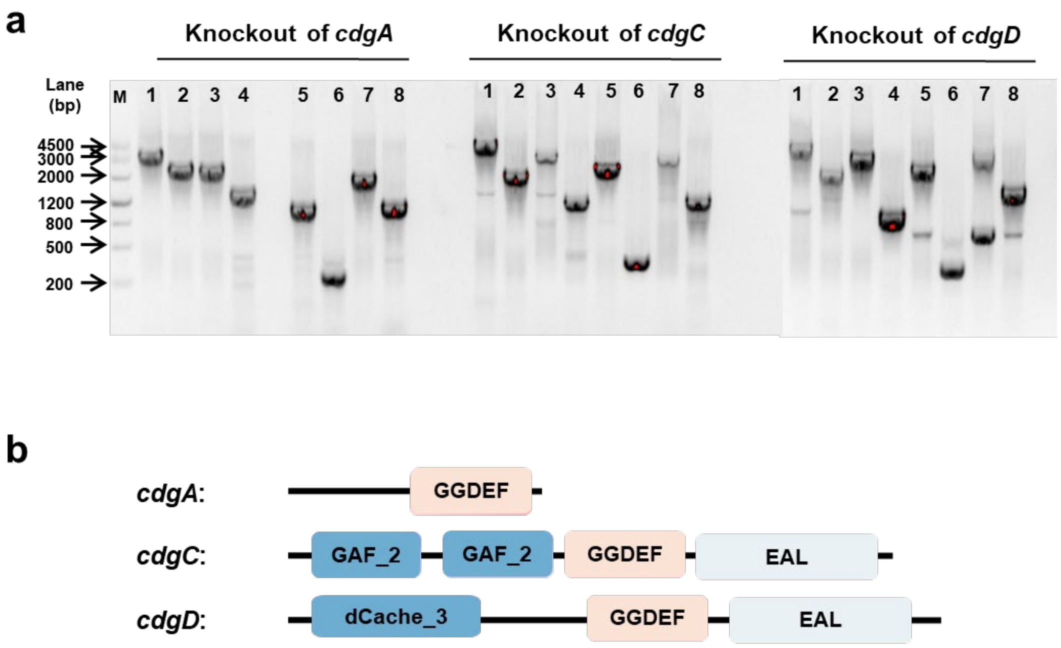

Supplementary Fig. 1. Construction of mutant strains. a. Identification of gene-deletion via PCR M was marker. The lanes 1, 3, 5, and 7 were the PCR products amplified using the DNA of wild-type strain, and the lanes 2, 4, 6, and 8 were the PCR products amplified using the DNA of deletion mutant. Lanes 1 and 2 showed DNA amplified using the primer pair L-F/L-R, lanes 3 and 4 after using L-F/S-R, lanes 5 and 6 using S-F/S-R, and lanes 7 and 8 using S-F/L-R. Each panel depicts DNA bands derived from the same experiment, and they were processed in parallel. **b.** The protein domains of the knockout of genes related to c-di-GMP biosynthesis: *cdgA*, *cdgC*, *cdgD*.

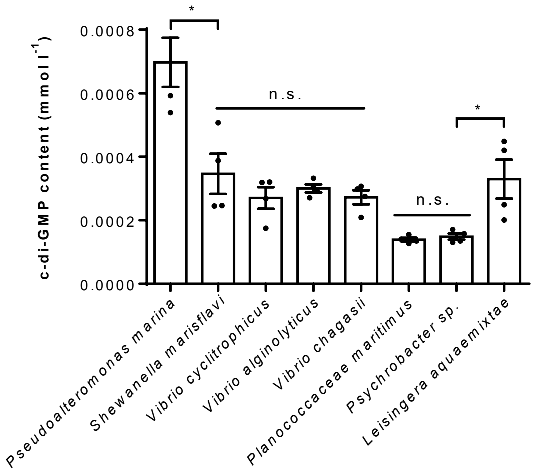

Supplementary Fig. 2. c-di-GMP content in the different extracts from bacteria at the same bacterial density.

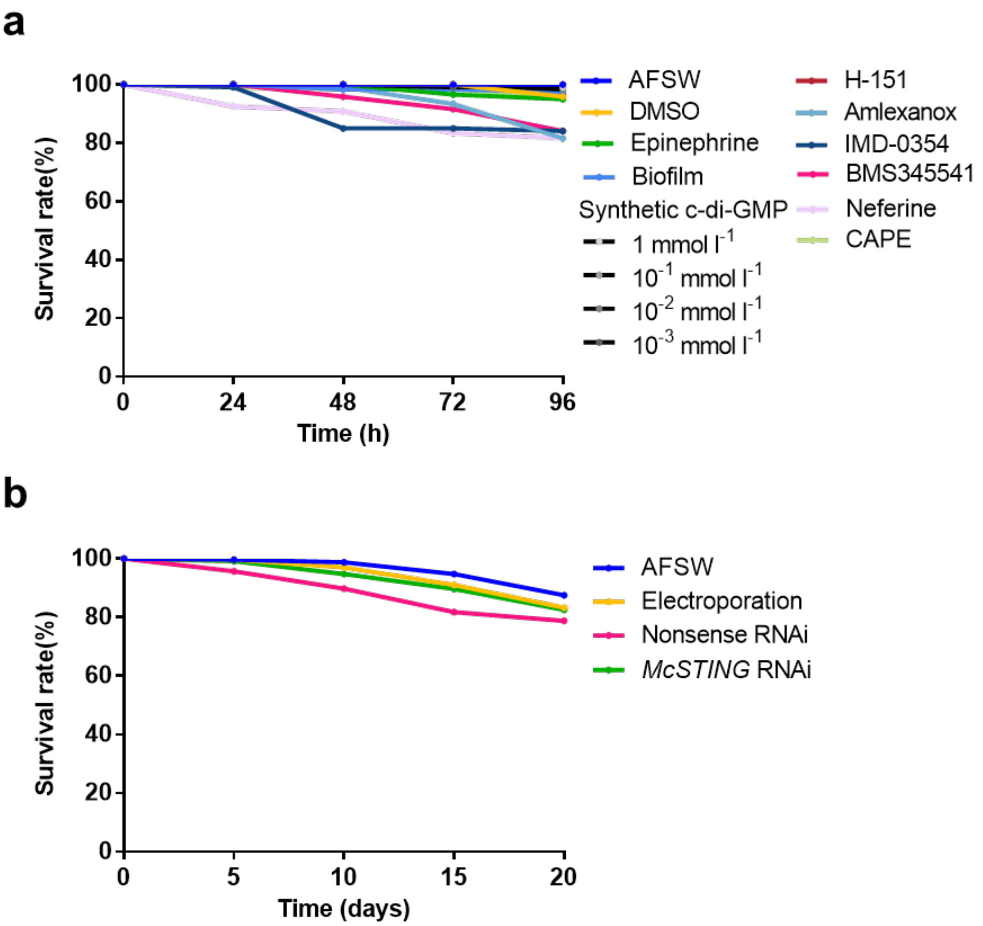

Supplementary Fig. 3. The survival rate of mussel larvae in pharmacological (a) and RNAi experiments (b).

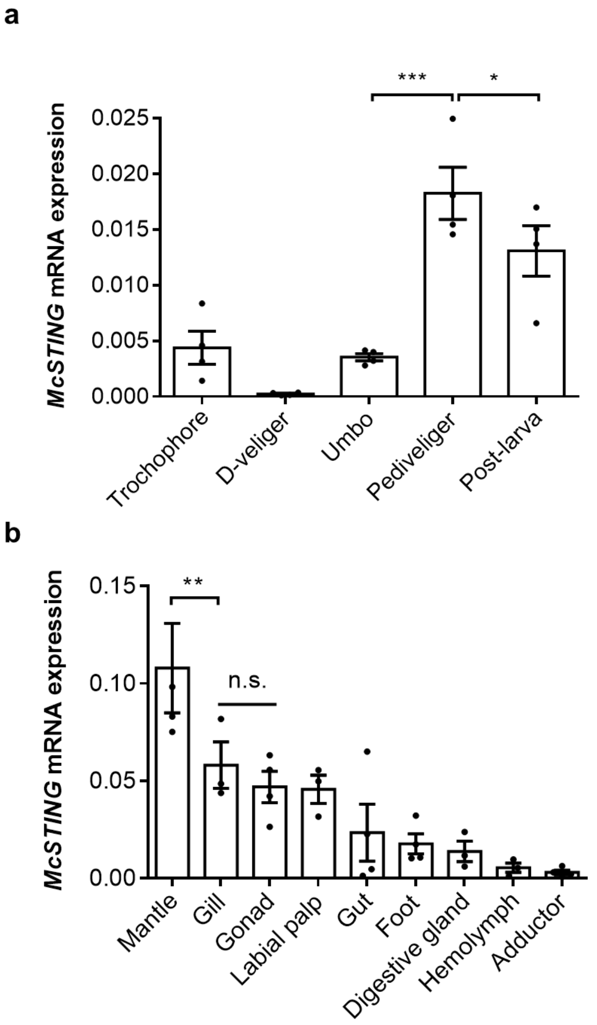

Supplementary Fig. 4. Expression of the *McSTING* gene during different developmental stages (a) and tissues (b) of *M. coruscus.* Error bars represent standard errors of 6 biological replicates. Statistical differences were discerned using the Student's t-test. *$p < 0.05$; **$p < 0.01$; ***$p < 0.001$. The same as below.

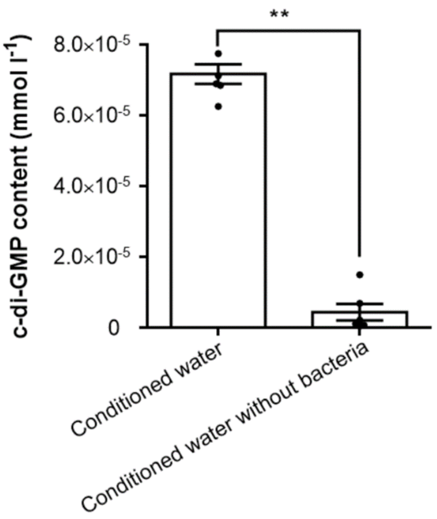

Supplementary Fig. 5. The c-di-GMP content in the conditioned water and in the conditioned water after filtering out the bacteria. Error bars represent standard errors of 6 biological replicates. Statistical differences were determined by Wilcoxon test for each pair.
